# Secure Grasping Detection of Objects in Stacked Scenes Based on Single-Frame RGB Images

**DOI:** 10.3390/s23198054

**Published:** 2023-09-24

**Authors:** Hao Xu, Qi Sun, Weiwei Liu, Minghao Yang

**Affiliations:** 1 School of Computer Science and Technology, Zhejiang Sci-Tech University, Hangzhou 310018, China; 202120503049@mails.zstu.edu.cn (H.X.); 202230603082@mails.zstu.edu.cn (W.L.); 2 The Research Center for Brain-Inspired Intelligence (BII), Institute of Automation Chinese Academy of Sciences (CASIA), Beijing 100190, China; mhyang@nlpr.ia.ac.cn

**Keywords:** secure grasping, object-stacking scene, grasping relationship, circular smooth label, object detection

## Abstract

Secure grasping of objects in complex scenes is the foundation of many tasks. It is important for robots to autonomously determine the optimal grasp based on visual information, which requires reasoning about the stacking relationship of objects and detecting the grasp position. This paper proposes a multi-task secure grasping detection model, which consists of the grasping relationship network (GrRN) and the oriented rectangles detection network CSL-YOLO, which uses circular smooth label (CSL). GrRN uses DETR to solve set prediction problems in object detection, enabling end-to-end detection of grasping relationships. CSL-YOLO uses classification to predict the angle of oriented rectangles, and solves the angle distance problem caused by classification. Experiments on the Visual Manipulate Relationship Dataset (VMRD) and the grasping detection dataset Cornell demonstrate that our method outperforms existing methods and exhibits good applicability on robot platforms.

## 1. Introduction

Robot grasping is a fundamental task in robot operation and lays the groundwork for completing complicated tasks. In the context of real grasping scenarios, complex scenes are common, and objects are frequently arranged in a stacked position, as seen in material handling and fruit sorting. If the grasped object is concealed by other objects, the object stack becomes unstable, and the rigid object may shatter. While it is intuitive for humans to select a stable object from a stack of objects, this poses a significant challenge for robots, since they solely rely on vision. Therefore, it is crucial for robots to make autonomous decisions to determine a secure grasping position to maintain the stability of the entire object stack.

The development of deep learning has led to two categories of vision-based robot grasping methods: six degrees of freedom (6DoF) grasping and 2D plane grasping [1]. Most 6DoF grasping methods require point clouds and intrinsic camera parameters to determine an object’s position, estimate the pose, and match the original object using templates [2,3], offering high precision but requiring significant computational resources. Some methods use local point clouds to accelerate computation, but this may lead to a loss of object edge features and incorrect candidate grasping positions [4]. Recent approaches have achieved positive results by optimizing the decision-making process and reducing interfaces to accelerate grasping position generation under 6DoF [5,6]. In scenarios where objects are on a plane and can only be grasped from one direction, 2D plane grasping is preferable. The main method for this is object detection through rotation, generating potential grasping positions in an image using data-driven convolutional neural networks [7]. However, the resulting grasp positions’ safety is not immediately apparent, and a scoring system is often utilized as a supplement to determine each grasping box’s security score [8]. This approach works well in specific scenarios but requires a large amount of data and lacks strong generalization capabilities. A solution to this problem is to assess the stacking relationship between objects before grasping, verifying the grasped object’s *z*-axis position in the final grasp using only the depth image, which reduces computational power. Traditional object stacking reasoning uses object pairwise pooling. However, this process is time-consuming, and it cannot consider global image information when multiple objects are in the image. Recently, transformers have been used to process images [9], allowing object detection to be transformed into an unordered set problem, providing the foundation for the object stacking relationship reasoning method proposed in this paper.

We propose a data-driven, multi-task secure grasping detection model in this paper which utilizes a single RGB frame to obtain global information by detecting object stacking relationships and grasping positions before obtaining the final secure grasping position via post-processing. The gripper we used in this paper is a parallel gripper. To preserve visual information within the image, we incorporate residual modules [10] into our Grasping Relationship Network (GrRN) for object stacking relationship detection, inspired by the network design of Adj-Net [11] and Deformable DETR [12]. Furthermore, we created a rotation-based object detection model called CSL-YOLO, using one-hot encoding, which is inspired by YOLOv5 6.0 [13] and circular smooth label (CSL) [14]. Our experiments, conducted using the Visual Manipulation Relationship Dataset (VMRD) [15] and Cornell [16], demonstrate that our proposed object stacking relationship detection and grasping position detection methods perform well. The primary contributions of this paper are as follows:

(1) Analyzing how to use an adjacency matrix to represent an object stack. We used the mathematical properties of the adjacency matrix and post-processing to obtain a secure grasp.
(2) Using the Hungarian algorithm of Deformable DETR [12] to generate predictions for object queries and corresponding relationships between objects, and then using this relationship and visual features learned by Encoder to generate an adjacency matrix. We analyzed the impact of multi-scale features and variable self-attention mechanisms on overall model performance. Adding residual modules between the original feature map and the output of Encoder provides adequate visual features for the input of the MLP that generates the adjacency matrix.
(3) Combining the CSL [14] idea with the one-stage object detection model YOLOv5 [13]. We demonstrated that angle prediction can be transformed from a regression problem to a classification problem using one-hot encoding and using Gaussian functions as a window function to improve the rationality of loss calculation.

This paper is organized as follows: Section 2 provides an overview of the research status of secure robot grasping. Section 3 details the use of the adjacency matrix to determine the optimal grasping object, the principles of predicting the adjacency matrix, and how to generate rotating grasping boxes. Section 4 demonstrates the performance of our method on a dataset, including testing its capabilities and presenting experimental results. Finally, Section 5 presents this paper’s conclusion.

## 2. Related Work

### 2.1. Object Detection

The accurate identification of object location and category within an image is crucial for successful stacking relationship detection. Predicting rotating rectangular boxes is a fundamental aspect of grasping detection and a part of object detection. Therefore, it is crucial to select an appropriate object detector. Recent advances in deep learning have led to the development of highly competent object detectors such as two-stage RCNN [17], Fast RCNN [18], and Faster RCNN [19], as well as one-stage SSD series [20], and YOLO series [13,21]. One-stage methods are faster than two-stage methods, but they have slightly lower accuracy. In recent years, the appearance of the transformer-based object detector, DETR [22], has become a new paradigm. DETR regards object detection as a set prediction problem, achieving end-to-end object detection and removing the artificially defined parts of traditional methods, allowing the adjacency matrix prediction problem to be implemented with an end-to-end network. The issue of weak performance on small objects and slow model convergence in DETR is resolved by Deformable DETR [12], which is selected as the backbone network. To enhance accuracy while maintaining real-time detection speed, YOLOv5 [13] employs mosaic augmentation, feature pyramid, and path aggregation methods, making it the ideal backbone network for grasp box detection.

### 2.2. Stacking Relationship Detection

Stacking relationships are crucial in identifying the optimal secure grasping method. Recently, VMRN [23], the first use of convolutional neural networks in stack relationship detection, was introduced by Zhang, who also published VMRD [15]. VMRN detects objects first and then uses convolutional operations on each object pair to predict the relationship between them. To expedite the time-consuming operation of convolution on each object pair, Park et al. [24] expanded the grasping information to 15 dimensions and utilized an optimized cross-scale YOLOv3 network FCNN to directly forecast object subcategories, significantly enhancing detection speed. Additionally, Chi et al. [25] affirmed the significance of spatial and semantic information of objects in inferring the stacking relationship and proposed the VSE model to improve the accuracy of stack relationship detection through encoded spatial and semantic information output by the bag-of-words model for object pair pooling. Furthermore, Tchuiev et al. [11] successfully solved the adjacency matrix prediction problem posed by the stacking challenge by leveraging end-to-end object detectors and proposed Adj-Net, which significantly improved the accuracy of detecting stacking relationships. This paper adopts Adj-Net and modifies the parts of the object detection and adjacency matrix prediction to improve the model detection performance of stacking relationships.

### 2.3. Grasping Detection

Traditional grasping methods typically utilize object texture, geometric shapes, and the tactile information of robotic hands for grasping detection [26,27]. In recent years, convolutional neural network-based grasping detection has grown increasingly popular. Guo et al. [28] introduced a hybrid depth structure that incorporates both visual and tactile sensors, leveraging tactile data to enhance visual information for more effective learning and ultimately improve grasping detection success rates. Similarly, Chu et al. [29] utilized Faster RCNN and a region proposal network to generate grasping boxes while converting the angle problem into a classification challenge with null hypotheses competition, resulting in significantly improved grasping box generation accuracy. Additionally, Dong et al. [30] proposed a two-stage method that entails first acquiring image mask features and subsequently generating grasping detection results by leveraging these mask features to mitigate the impact of cluttered background information on grasping detection accuracy. In recent years, one-stage object detection and rotation box detection methods have developed rapidly, and the proposed CSL [14] provides a good solution for angle classification problems and can adapt to different object detectors.

## 3. The Method of Grasping in Stacked Scenes

**Figure 1:**
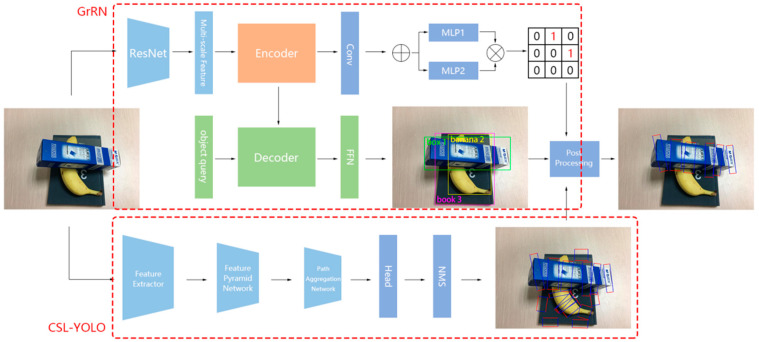
The model’s overall structure comprises the proposed grasping relationship detection network at the top, which employs Deformable DETR for object detection and generates the adjacency matrix by multiplying two feature matrices. The bottom part is the proposed rotation box detection method. Subsequently, the final grasping results are obtained via post-processing.

Our proposed multi-task model comprises two components: the Grasping Relationship Network (GrRN) and the CSL-YOLO network. GrRN employs a multi-scale transformer to detect grasp sequences, while CSL-YOLO is an improved YOLOv5 network that utilizes CSL. The outputs of both tasks are then subjected to a post-processing operation to determine the suggested grasping positions. The input of the model is an RGB image, and the output is the secure grasping position in a single RGB frame. Figure 1 provides an overview of the overall model structure.

### 3.1. Initialization with Adjacent Matrix

In complex scenes, objects are frequently stacked. We represent each object as a node, and the relationship between two stacked objects as a weighted edge. Thus, any object stack can be represented by a weighted directed graph $G≜\left(V,E,W\right)$ with $N_{V}$ nodes 𝓋∈V and $N_{E}$ edges ϵ∈E, where each edge has a weight ω∈W. For two objects, o1 and o2, if o1 directly overlaps object o2, an edge $\epsilon_{o1\to o2}$ is formed, with the weight ω representing the probability of its existence. In the dataset, ω = 1, whereas during prediction, the value of ω ranges between 0 and 1.

Our primary objective is to predict the weighted directed graph G, which can be represented by an adjacency matrix A in data structures:

(1) $$A=\left[\begin{array}{cccc} 0 & \omega_{12} & \cdots & \omega_{1V}\\ \omega_{21} & 0 & \cdots & \omega_{2V}\\ \vdots & \vdots & \ddots & \vdots \\ \omega_{V1} & \omega_{V2} & \cdots & 0 \end{array}\right]$$

The adjacency matrix A represents the stacking relationship between objects in the object stack, and its size is $N_{V}\times N_{V}$. A diagonal element in A must be 0, since an object cannot overlap itself. The element $\omega_{\mathrm{ij}}$ in the row i and column j of A represents the probability of the existence of edge $\epsilon_{\mathrm{oi}\to \mathrm{oj}}$. Since the object detection results’ order may be uncertain (i.e., ${\mathrm{index}}_{\mathrm{pre}}$ and ${\mathrm{index}}_{\mathrm{origin}}$ may not correspond), the adjacency matrix $A_{\mathrm{gt}}$ is not unique and is determined by the actual order of the object detection results. We can calculate the $A_{\mathrm{gt}}$ using a unit matrix $E_{\mathrm{change}}$ after row and column transformations based on the relationship between ${\mathrm{index}}_{\mathrm{pre}}$ and ${\mathrm{index}}_{\mathrm{origin}}$, as follows:

(2) $$E_{\mathrm{change}}=\mathrm{getChange}\left({\mathrm{index}}_{\mathrm{pre}},{\mathrm{index}}_{\mathrm{origin}}\right)$$

(3) $$A_{\mathrm{gt}}=A_{\mathrm{origin}}\cdot E_{\mathrm{change}}$$

The dataset predefines ${\mathrm{index}}_{\mathrm{origin}}$ and $A_{\mathrm{origin}}$, while ${\mathrm{index}}_{\mathrm{pre}}$ is determined through the Hungarian algorithm and post-processing during object detection. To predict the adjacency matrix A, we multiply a matrix ${\mathrm{adj}}_{1}$ with $N_{V}$ rows and a matrix ${\mathrm{adj}}_{2}$ with $N_{V}$ columns, resulting in the predicted value of matrix A, denoted as $A_{m}$.

**Figure 2:**
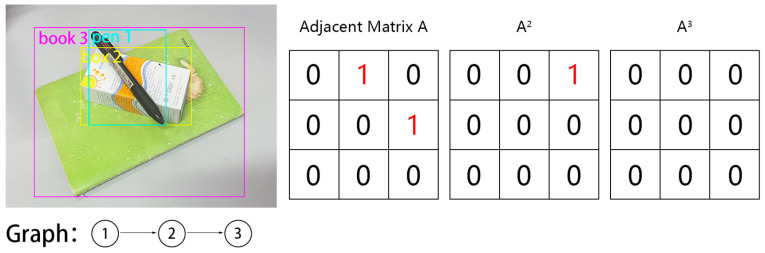
The left-hand side of the figure presents a stack of objects and its directed graph, while the right-hand side shows the corresponding adjacency matrix and its power. To calculate the secure grasping, we utilize the n-th power of the adjacency matrix. Elements of the matrix’s i-th row and j-th column denote the probability of covering.

To achieve secure grasping, the n-th power of the adjacency matrix A can be used. The matrix power calculation can determine if there are still objects between two objects, thus obtaining the uncovered objects in the object stack. As demonstrated in Figure 2, we consider an object stack with object o1 covering object o2 and object o2 covering object o3. We can obtain the adjacency matrix A for this object stack. For elements $\omega_{\mathrm{ij}}$ in the n-th power matrix $A^{n}$ of A where $\omega_{\mathrm{ij}}$ = 1, there are (n − 1) objects between object $o_{i}$ and object $o_{j}$. When $A^{n}\left(n\neq 1\right)$ is a matrix of all zeros, $\omega_{\mathrm{ij}}$ values equal to 1 in $A^{n-1}$ signify that object $o_{i}$ can be grasped safely. When A consists entirely of zeros, it implies that every object can be grasped safely.

### 3.2. GrRN

**Figure 3:**
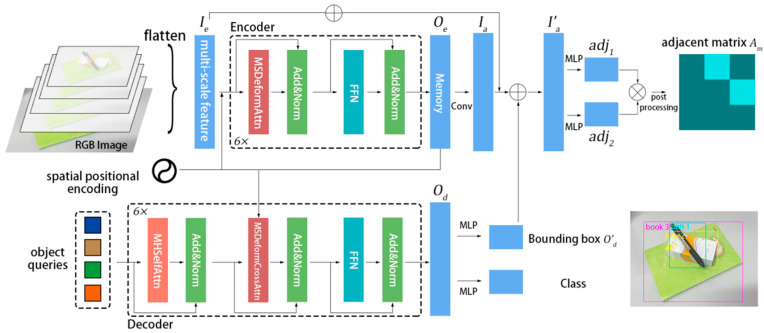
The network architecture of GrRN. The image generates multi-scale features after going through a feature extractor, and then obtains object detection results through Deformable DETR. After visual enhancement, the adjacency matrix is predicted and the dark portion in the matrix represents 0, while the bright portion represents 1.

After observing the impressive capabilities of end-to-end object detection models such as DETR [22] in resolving matrix prediction problems, notably the inspiring results of Adj-Net [11], we aimed to incorporate these findings into our research. Traditional solutions to the stacking prediction problem involve multi-stage methods requiring object detection to establish the point set V of a directed graph, which is then matched to obtain the edge set E and probability set W for the existence of edges. Consequently, the adjacency matrix prediction problem is categorized as a set prediction problem. DETR [22] regards object detection as a set prediction problem, which can directly obtain the node set V of the directed graph without requiring post-processing operations, providing great convenience for predicting the weighted edge set E in subsequent steps. We based our experiments on Deformable DETR [12], which resolves the issues of sluggish convergence and poor performance on small objects found in DETR. The GrRN is presented in Figure 3.

GrRN takes RGB images as its input and outputs predictions for object detection and the corresponding adjacency matrix. The model initially extracts multi-scale features $I_{e}$ (input of Encoder) of the image using a feature extractor (ResNet50 in this paper). The number of scales is 4, consistent with Deformable DETR [12]. The dimensions of $I_{e}$ are e×h. Six multi-head self-attention modules utilize $I_{e}$ to generate $O_{e}$ (output of Encoder), with the dimensions of e×h. Decoder takes the object query and $O_{e}$ as inputs. The dimensions of the object query are q×h. $O_{d}$ is the output of Decoder, with dimensions q×h. Feeding the output of Decoder through a feedforward network generates the detection results for bounding boxes (${O\prime }_{d}$) and class detections. The dimensions of ${O\prime }_{d}$ are q×4, while the dimensions of class detections are $q\times \left(N_{\mathrm{class}}+1\right)$, where 1 denotes the absence of an object. To enhance the visual information of the features, the model connects $I_{e}$ residually with $O_{e}$ and remodels it into h×1×e. We utilize a convolution operation to alter the depth and obtain the feature map $I_{a}$, with the dimensions of h×1×q. Subsequently, it is resized to q×h. Merging ${O\prime }_{d}$ and $I_{a}$ yields ${I\prime }_{a}$ with the dimensions of $q\times \left(h+4\right)$. The model processes ${I\prime }_{a}$ through two independent MLP operations that do not alter its dimensions. These operations yield two matrices, ${\mathrm{adj}}_{1}$ and ${\mathrm{adj}}_{2}$, with the dimensions of $q\times \left(h+4\right)$. The matrices are then used for calculating the adjacency matrix. The model multiplies ${\mathrm{adj}}_{1}$ and ${\mathrm{adj}}_{2}^{T}$, and the result goes through a sigmoid operation to yield the preliminary prediction for the adjacency matrix, $A_{p}$. The size of $A_{p}$ is q×q. After finding the result of the Hungarian matching, the indices $i_{1},i_{2},I,i_{m}$ of the objects from q are generated. The corresponding rows and columns are then extracted from $A_{p}$ to obtain the final adjacency matrix, $A_{m}$.

We attempted to use Decoder’s output, $O_{d}$, to predict the adjacency matrix. However, the utilization of $O_{e}$ produced much better results. DETR suggests that Decoder has the capability to learn more about the object’s boundary information while Encoder retains more visual information about the object. Given the importance of visual information in determining whether objects are stacked, we postulate that using Encoder’s output to predict the adjacency matrix is more appropriate.

Due to the increased ability of the model to predict adjacent matrices, we need to consider the loss of predicting adjacent matrices when calculating the loss. The loss of the entire model can be divided into two parts: bipartite matching loss and model optimization loss. Since the prediction of the adjacent matrix is made after bipartite matching, the loss of bipartite matching remains the same and is not modified, just like in DETR. For the model optimization loss, we consider it from the following perspectives.

The initial aspect to consider is the classification loss, which we evaluate using the cross-entropy loss. The formula for the cross-entropy loss is as follows:

(4) $$L_{\mathrm{class}}=-\sum_{p\in P}^{}\sum_{c=1}^{N_{\mathrm{class}}+1}{𝓌}_{c}\cdot y_{\mathrm{gt}}\left(c_{p}\right)\cdot \log\left(y_{\mathrm{pre}}\left(c_{p}\right)\right)$$

where p∈P represents all proposed boxes obtained through bipartite graph matching, $N_{\mathrm{class}}$ is the number of classes in the dataset, including the “no object” class represented by 1. Since the occurrence of the “no object” class is greater than other object classes in practical detection tasks, we assign a weight ${𝓌}_{c}$ to each class during the calculation of classification loss. The weight assigned to the “no object” class is 0.01, compared to 1 assigned to other classes. We use $y_{\mathrm{gt}}\left(x\right)$ and $y_{\mathrm{pre}}\left(x\right)$ to represent the true and predicted class values of the ground truth box corresponding to the predicted box x, respectively.

For the bounding boxes, we use l1 loss and GIoU loss based on the recommendation of DETR. While l1 loss is sensitive to the size of the bounding box, it does not always precisely represent the distance between the predicted and ground truth boxes. Therefore, we use the GIoU loss as an auxiliary measure. The formula for both losses is as follows:

(5) $$L_{l1}=-\sum_{p\in P}^{}\left|B_{\mathrm{gt}}\left(p\right)-B_{\mathrm{pre}}\left(p\right)\right|$$

(6) $$L_{\mathrm{GIoU}}=-\sum_{p\in P}^{}\left(1-\left(\frac{S_{\mathrm{gt}}\cap S_{\mathrm{pre}}}{S_{\mathrm{gt}}\cup S_{\mathrm{pre}}}-\frac{S_{c}-S_{\mathrm{gt}}\cup S_{\mathrm{pre}}}{S_{c}}\right)\right)$$

When calculating the l1 loss, we measure the distances between the predicted and actual values of cx, cy, w, and h independently. $S_{\mathrm{gt}}$ and $S_{\mathrm{pre}}$ represent the surface areas of the ground truth box and predicted box, respectively. The minimum bounding box that encompasses both the ground truth and predicted boxes is represented by c.

The adjacency matrix $A_{m}$ is mostly sparse, with the majority of the values being 0. We adopt the binary cross-entropy loss function, from Adj-Net, to calculate the loss. In comparison with l1 and l2 losses, binary cross-entropy loss can effectively penalize incorrect 0 values, resulting in a faster model convergence speed. The formula for binary cross-entropy loss is as follows:

(7) $$L_{\mathrm{adj}}=-\sum_{i,j\in A_{m}}^{}\left(A_{\mathrm{ij}}^{\mathrm{gt}}{\mathrm{logA}}_{\mathrm{ij}}^{\mathrm{pre}}+\left(1-A_{\mathrm{ij}}^{\mathrm{gt}}\right)\log\left(1-A_{\mathrm{ij}}^{\mathrm{pre}}\right)\right)$$

The ultimate loss for the GrRN model is a weighted sum of all losses mentioned above:

(8) $$L_{\mathrm{total}}=\lambda_{\mathrm{class}}L_{\mathrm{class}}+\lambda_{l1}L_{l1}+\lambda_{\mathrm{GIoU}}L_{\mathrm{GIoU}}+\lambda_{\mathrm{adj}}L_{\mathrm{adj}}$$

where all λ values are hyperparameters.

### 3.3. CSL-YOLO

In the context of 2D robotic grasping, rotated rectangles are commonly used to represent the area in which the robotic arm should grasp. We implemented modifications to the long-side representation method to suit the field of robotic grasping, resulting in the grasp-side representation method. This approach is denoted by $\left(x,y,h,w,\theta \right)$, where x and y denote the central coordinates of the rectangle, h indicates the length of the grasping side, w refers to the distance between the robotic fingers’ openings, and θ has the range $\left[-90{}^{\circ},90{}^{\circ}\right)$. Due to the limitations of annotation tools, the available angle values in the dataset include {−90°, −89°, ..., 88°, 89°}.

**Figure 4:**
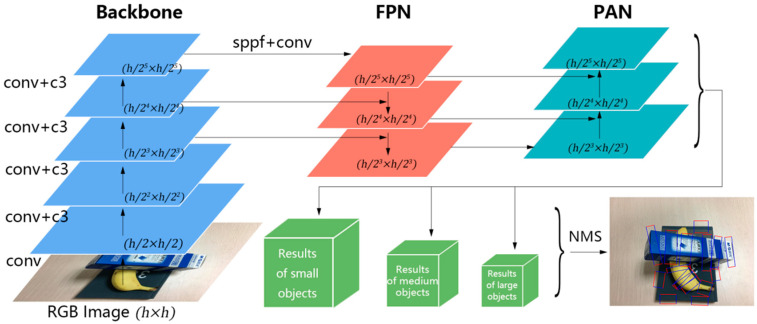
The network architecture of CSL-YOLO. The input of the network is an RGB image, and the output is a rotated grasping box.

To predict the grasp boxes, we based our work on YOLOv5 and developed CSL-YOLO, which is built upon the CSL. The input of CSL-YOLO is an RGB image, and the output of the model is all potential grasp boxes in the image. Like YOLOv5, CSL-YOLO consists of a backbone, neck, and head. The structure of the model is shown in Figure 4.

RGB images are first zero-padded so that their width and height are the same as each other, then resized to h×h. The backbone uses these resized images to extract visual features, reducing the image’s width and height by half as it passes through successive feature layers. The lower convolutional layers learn visual features related to object contours, while higher layers extract more semantic features. The Feature Pyramid Network (FPN) is used to transmit strong, semantic features from the higher layers to the lower layers, while the Path Aggregation Network (PAN) transmits positional features from the lower layers to the higher layers. The head generates the final three output feature maps, which predict objects at three different scales. The high-resolution feature map is best suited for small objects, whereas the low-resolution feature map is better for larger objects. During training, the object’s center point position is used to calculate the loss. Non-Maximum Suppression (NMS) is used to avoid the over-representation of objects in the output.

To facilitate angle prediction in YOLOv5, we referred to CSL and treated angle prediction as a classification problem instead of a regression one. Unlike regression, the classification problem can address the boundary problem. Angles exhibit periodicity, and −90° and 89° are equivalent. The loss between these angles ought to be minimal, but regression will yield high loss values. Classification considers every prediction, right or wrong, to be equal, eliminating the boundary problem. Nonetheless, classification fails to provide information about the distance between two angles. In fact, angles close to the true angle are admissible, and the model should minimize the loss for such angles. CSL replaced the true label in the cross-entropy loss function with $\mathrm{CSL}\left(x\right)$. This replacement allows the model to penalize predictions closer to the true angle less, improving the accuracy of angle prediction. The formula to compute $\mathrm{CSL}\left(x\right)$ is:

(9) $$\mathrm{CSL}\left(x\right)=\left\{\begin{array}{cc} g\left(x\right), & \theta -r<x<\theta +r\\ 0, & \mathrm{otherwise} \end{array}\right.$$

where x represents the predicted angle by the model, θ represents the actual angle of the grasping box, $g\left(x\right)$ is the window function, and r is the window radius. We apply a penalty that decreases as the predicted angle falls within the window radius of θ. Based on the results of our ablation experiments, we defined r as 6. After replacing the true label, the formula for the new loss function is as follows:

(10) $$L_{\theta }=-\sum_{i}^{}\sum_{x=-90}^{89}\mathrm{CSL}\left(x\right)\cdot \log\left(x\right)$$

Since there are no categories for grasp boxes in this study, category loss is not necessary. The other loss functions remain unmodified, and thus the final loss function of the CSL-YOLO model is:

(11) $$L_{\mathrm{total}}=\lambda_{\mathrm{bbox}}L_{\mathrm{bbox}}+\lambda_{\mathrm{conf}}L_{\mathrm{conf}}+\lambda_{\theta }L_{\theta }$$

where all λ values are hyperparameters.

## 4. Experiment and Result Analysis

This chapter presents experimental results for GrRN and CSL-YOLO, along with an investigation of the impact of grasping in a real-world scenario. The proposed models were implemented using the PyTorch 1.12.1 framework and trained and tested using an NVIDIA Tesla V100 with 16 G memory. To verify the grasping algorithm in a real-world stacking scenario, we utilize a 4DoF Kinova gen2 robotic arm and an Intel Real Sense2 depth camera.

### 4.1. Experimental Setup for GrRN

The proposed grasp relationship detection method was trained and validated on the VMRD [15] using a 9:1 ratio for the training and validation sets, which consisted of 4233 images, and a test set with 450 images. Due to the high computational expenses of the multi-task secure grasping method, we employed ResNet50 as the feature extractor, which has relatively few parameters and low computation costs. The model specifications were set as follows: h=256 for number of hidden dimensions, eight for the number of heads in the variable transformer module, four for the number of reference points in the variable self-attention, six for the number of modules in Encoder and Decoder, and 300 for the quantity of object queries. The convolution kernel size that changed dimensions was 1×1×300. The two MLPs that predicted the adjacency matrix had the following specifications: the number of input dimensions was h+4=260, the number of hidden dimensions was 260, and the number of output dimensions was 260. They had three hidden layers. The AdamW optimizer was used to train the network. During training, the adjacency matrix prediction part was frozen at first, and the object detection part was trained for 300 epochs utilizing the COCO dataset at a learning rate of 0.001. Subsequently, the whole network was trained on VMRD for 500 epochs at a learning rate of 0.0001.

### 4.2. Experimental Results of GrRN

Our method’s effectiveness was evaluated using the VMRD, and its performance was compared to three of the most stacked object detection algorithms—VMRN, VSE, and Adj-Net. We utilized the detection results from Adj-Net and considered them accurate under the following circumstances:

- For objects i and j where i is placed on j, $\mathbb{P}\left(\exists \epsilon_{i\to j}\right)>0.5$ and $\mathbb{P}\left(\exists \epsilon_{i\to j}\right)>\mathbb{P}\left(\exists \epsilon_{j\to i}\right)$.

- For objects i and j that have no direct relationship, $\mathbb{P}\left(\exists \epsilon_{i\to j}\right)<0.5$ and $\mathbb{P}\left(\exists \epsilon_{j\to i}\right)<0.5$.

In the field of object detection, several concepts are used, including true positive (TP), false positive (FP) for incorrect predictions, true negative (TN), and false negative (FN) for missed detection. Our evaluation of the model’s object detection performance is based on two metrics: Object Recall (OR) and Object Precision (OP). The formulas for calculating OR and OP are:

(12) $$\mathrm{OR}=\frac{\mathrm{TP}}{\mathrm{TP}+\mathrm{FN}}$$

(13) $$\mathrm{OP}=\frac{\mathrm{TP}}{\mathrm{TP}+\mathrm{FP}}$$

When detecting grasping relationships, we utilize the standard measures of true positive (TP), false positive (FP) for incorrect predictions, true negative (TN), and false negative (FN) for missed detection, following the practices of object detection. To evaluate our model’s performance, we use three metrics:

- Relationship Recall (RR): The number of correctly detected relationships divided by the total number of correct stacking relationships.
- Relationship Precision (RP): The quantity of correctly predicted relationships divided by the total quantity of detected relationships. If the tuple $\left(o_{i},R_{\mathrm{ij}},o_{j}\right)$ is correct, the detected relationship is considered correct, where $o_{i}$ represents the i-th object and R represents the relationship between the two objects in the indices.
- Image Accuracy (IA): In the test set, RR and RP are both 100% for all the existing objects in the image. The notation IA-x represents the presence of x objects in the image.

**Figure 5:**
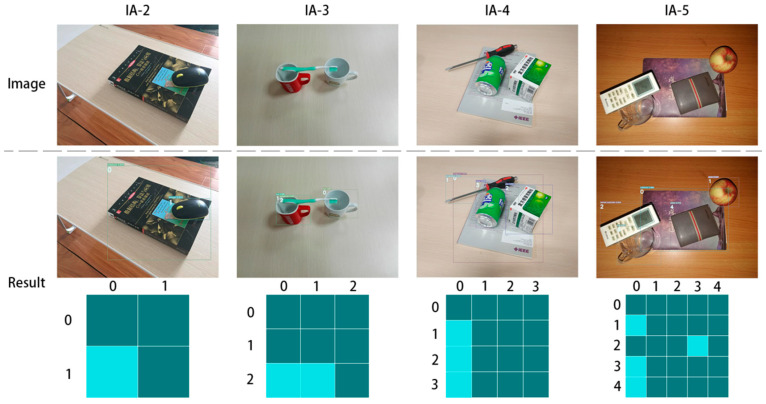
Stacking relationship detection results of our methods on Visual Manipulation Relationship Dataset. The first row of images contains stacks of objects with varying numbers. The second row of images displays the results of the object detection. The third row of images shows the predicted results of the adjacency matrix.

Figure 5 shows some detection results of our methods on VMRD. One image was chosen from each of IA-2 to IA-5 for display. The top row displays the original images, while the second row displays the results of object detection, including bounding boxes, categories, confidence scores, and object indexes. The bottom row shows the predicted adjacency matrices, with dark squares indicating the value of 0, and light squares indicating the value of 1.

The comparison of the object detection results with other models is shown in Table 1, and as ResNet50 was the feature extractor we employed, Adj-Net utilized the same feature extractor. Our method was more effective than current state-of-the-art approaches. The more advanced deep learning becomes, the better object detectors perform, resulting in fewer false positives and negatives, aiding in the inference of object stacking relationships.

The comparison of the grasping detection results with other models is shown in Table 2. Our method exhibits superior performance as compared to the current best method. The object detection process now benefits from an improved performance, which leads to the easier detection of objects in the image. Consequently, the efficacy of the adjacency matrix detection also increases. The existing techniques for predicting object stacking relationships necessitate pooling convolution operations between object pairs, allowing predictions for only two objects at a time. This process proves to be time-consuming with an increased number of objects in the input image. However, the advent of end-to-end object detection facilitates the prediction of the stacking relationships for all objects simultaneously.

The current study focuses on images that contain between two and five objects within the VRMD. We assessed the efficacy of various models under different object conditions, as presented in Table 3. Our method outperformed all the other considered techniques overall. Notably, precision levels decrease significantly as the number of objects within the image increases and the inherent object relationships become more complex.

Table 4 exhibits the comparison of results obtained from GrRN-DETR (with DETR as a backbone network) and GrRN-Decoder (with Decoder output) in predicting the adjacency matrix. The effectiveness of DETR as a backbone network is compromised by its inability to correctly identify smaller objects, sensitivity to convergence time, and inferior object detection performance. As a result, the ability of the DETR-based model to predict the adjacency matrix is also compromised. The GrRN-Decoder model, on the other hand, lacks visual information, impeding the convergence of the adjacency matrix prediction component.

### 4.3. Experimental Setup for CSL-YOLO

For this study, we utilized the VMRD and the Cornell datasets with a total of 5568 images, distributed in a 8:1:1 ratio for training, validation, and test sets, respectively. The effectiveness of different window sizes {2, 4, 6, 8} was tested using the Gaussian function as the window function. Training incorporated a warm-up strategy while disabling mosaic data augmentation, with the application of Adam optimization at a learning rate of 0.0001.

### 4.4. Experimental Results for CSL-YOLO

To assess the efficacy of grasping detection, the rectangle metric was employed in this study. A predicted grasping was considered valid under two conditions: (1) the predicted grasping box has a rotation angle that varies by no more than 30 degrees from the true box, and (2) the Jaccard index $J\left(A,B\right)=\left|A\cap B\right|/\left|A\cup B\right|$ between the predicted grasping box A and the true box B is greater than 25%.

We use Image-wise (IW) and Object-wise (OW) to evaluate the performance of the model. The definitions of IW and OW are as follows:

- IW: The entire dataset is shuffled and randomly divided into training and test sets to test the model’s generalization ability for previously seen objects when they appear at new positions and rotation angles.
- OW: The dataset is divided by object instance, and the objects in the test set have not appeared in the training set before, to test the model’s generalization ability for unseen objects.

**Figure 6:**
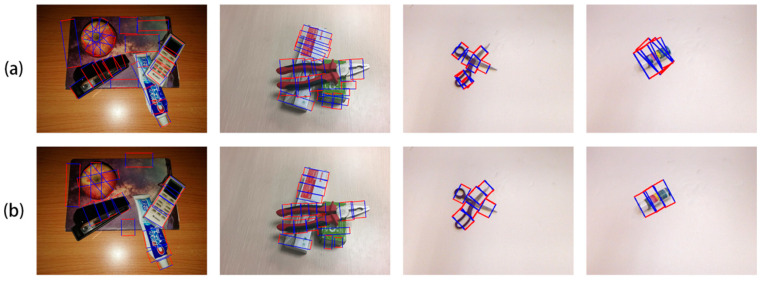
Grasping detection on Visual Manipulation Relationship Dataset and Cornell. (**a**) is the ground truth, and (**b**) is the result detected by our method.

Our method’s grasping detection results on the VMRD and Cornell datasets are presented in Figure 6. The ground truth data from the original datasets are displayed in the first row, with our detection results in the second row.

The study began by evaluating the model’s efficacy under different window sizes relative to traditional approaches. A summary of the outcomes, presented in Table 5, indicated superior grasping detection capabilities for the model when a window size of six was used. Notably, the window size directly affects the model’s grasp detection ability: undersized windows may exclude some grasping boxes that should be identified, impairing the model’s ability to attain local optima, whereas oversized selections may produce partially accurate outputs that affect model judgments. Evidently, the IW value surpassed the OW value as the model’s error rate increased while evaluating objects not represented in the dataset.

### 4.5. Experiments in Real-World Scenarios

**Figure 7:**
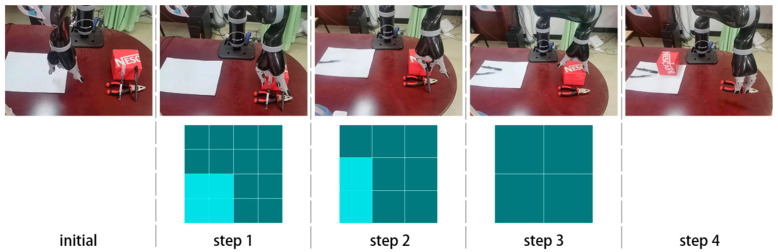
Robotic arm grasping in a real-world scenario. In the matrix, the dark portion represents 0, while the light portion represents 1.

This study utilized various objects in real-world scenarios to form distinct object stacks. RGB images, obtained through depth cameras, underwent object detection, adjacency matrix prediction, and grasping detection. Grasping boxes were selected based on the coefficient of overlap, $K\left(o,g\right)=\left(S_{o}\cap S_{g}\right)/S_{g}$ greater than 0.5, where o refers to the object box, g to the grasping box, and S to the box area. The grasping box closest to the center point of the object box was selected for use as the final grasping object for the robot arm. Grasping is then performed using the depth image information. Figure 7 depicts a specific grasping experiment where the robotic arm needs to move the objects on the right stack to the designated position on the left. The grasping process of the robotic arm is shown in the first row, while the predicted results of the adjacency matrix before each grasp is shown in the second row.

## 5. Conclusions

This paper proposes a multi-task deep neural network framework as a solution to the challenge of secure grasping in stacking scenarios. The framework commences with executing two pre-tasks: stacking relationship detection and grasping detection, before proceeding to the secure grasping task through post-processing. At first, the stacking relationship detection model detects objects within the RGB images, then predicts the object stack’s adjacency matrix by merging visual detection and object detection information. The adjacency matrix is then utilized to select an object in the current grasp sequence. A visual information enhancement module was employed to boost model efficiency. The grasping detection model utilizes a one-stage object detection model to predict the grasping box, classification techniques to solve the angle prediction problem, and the CSL methodology to boost the model’s ability to judge angle distance. On the VMRD and the Cornell datasets, our approach outperformed traditional methods and achieved secure grasping in real-world scenarios. In the future, there will be further improvements aimed at accelerating model prediction accuracy and speed.

## Figures and Tables

**Table 1:** Results of object detection from different models.

Model	OR (%)	OP (%)
VMRN	86.0	88.8
VSE	89.2	90.2
Adj-Net	90.1	93.5
Ours	91.9	94.8

**Table 2:** Results of grasp relationship from different models.

Model	RR (%)	RP (%)	IA (%)
VMRN	86.0	88.8	67.1
VSE	-	-	73.7
Adj-Net	88.9	91.5	74.4
Ours	91.2	93.1	78.0

**Table 3:** Results of grasp relationship IA-x from different models.

Model	Total (%)	IA-2	IA-3	IA-4	IA-5
VMRN	67.1	57/65	134/209	60/106	51/70
VSE	73.7	57/65	146/209	75/106	54/70
Adj-Net	74.4	56/65	155/209	74/106	50/70
Ours	78.0	60/65	160/209	79/106	52/70

**Table 4:** Results of different ways to calculate adjacent matrix.

Model	OR (%)	OP (%)	RR (%)	RP (%)	IA (%)
GrRN-DETR	86.1	88.7	86.5	89.7	71.2
GrRN-Decoder	92.3	95.2	54.4	59.6	30.3
GrRN	91.9	94.8	91.2	93.1	78.0

**Table 5:** Results of grasping detection from different models and window size.

Model	Grasp Detection Accuracy (%)	
	IW	OW
Guo	93.2	89.1
Chu	96.0	96.1
Dong	96.4	95.5
CSL-YOLO (r=2)	95.1	94.9
CSL-YOLO (r=4)	97.7	97.2
CSL-YOLO (r=6)	98.0	97.4
CSL-YOLO (r=8)	97.3	97.1

## Data Availability

The data are unavailable due to privacy restrictions.
